# Supplementary material for: Identification and characterization of epicuticular proteins of nematodes sharing motifs with cuticular proteins of arthropods
Source: PLoS One. 2022 Oct 27;17(10):e0274751. doi: 10.1371/journal.pone.0274751 (PMC9612446; doi:10.1371/journal.pone.0274751)
Supplement: S1 Fig — (DOCX) [file pone.0274751.s001.docx]

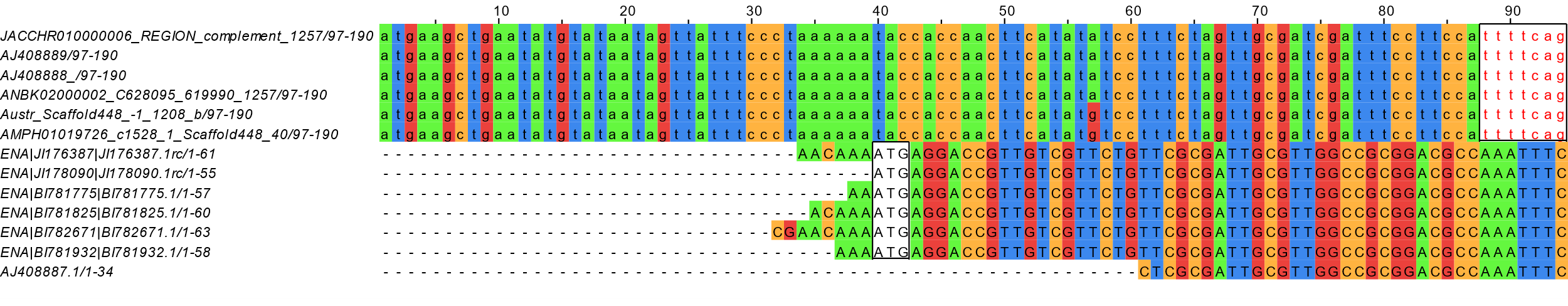


**S1 Figure.** Out of the 44 sequences listed in S1 Table, the 5’ regions of six *Asu-epic-1* genomic (small letters) and seven cDNA/TSA sequences (capital letters) are aligned. The genomic sequences located upstream of the splice acceptor site and the 5' unrepeated region of the cDNA/TSA sequences are completely different. The splice acceptor site (red letters) upstream of the repeated region in the genomic sequences indicates that the 5' unrepeated region represents a part of an intron. In the cDNA/TSA sequences, the 5’ unrepeated part is the first exon of 55 bases of *Asu-epic-1*, starting with ATG (S2 Figure).
