# Supplementary material for: Identification and characterization of epicuticular proteins of nematodes sharing motifs with cuticular proteins of arthropods
Source: PLoS One. 2022 Oct 27;17(10):e0274751. doi: 10.1371/journal.pone.0274751 (PMC9612446; doi:10.1371/journal.pone.0274751)
Supplement: S2 Fig — (DOCX) [file pone.0274751.s002.docx]

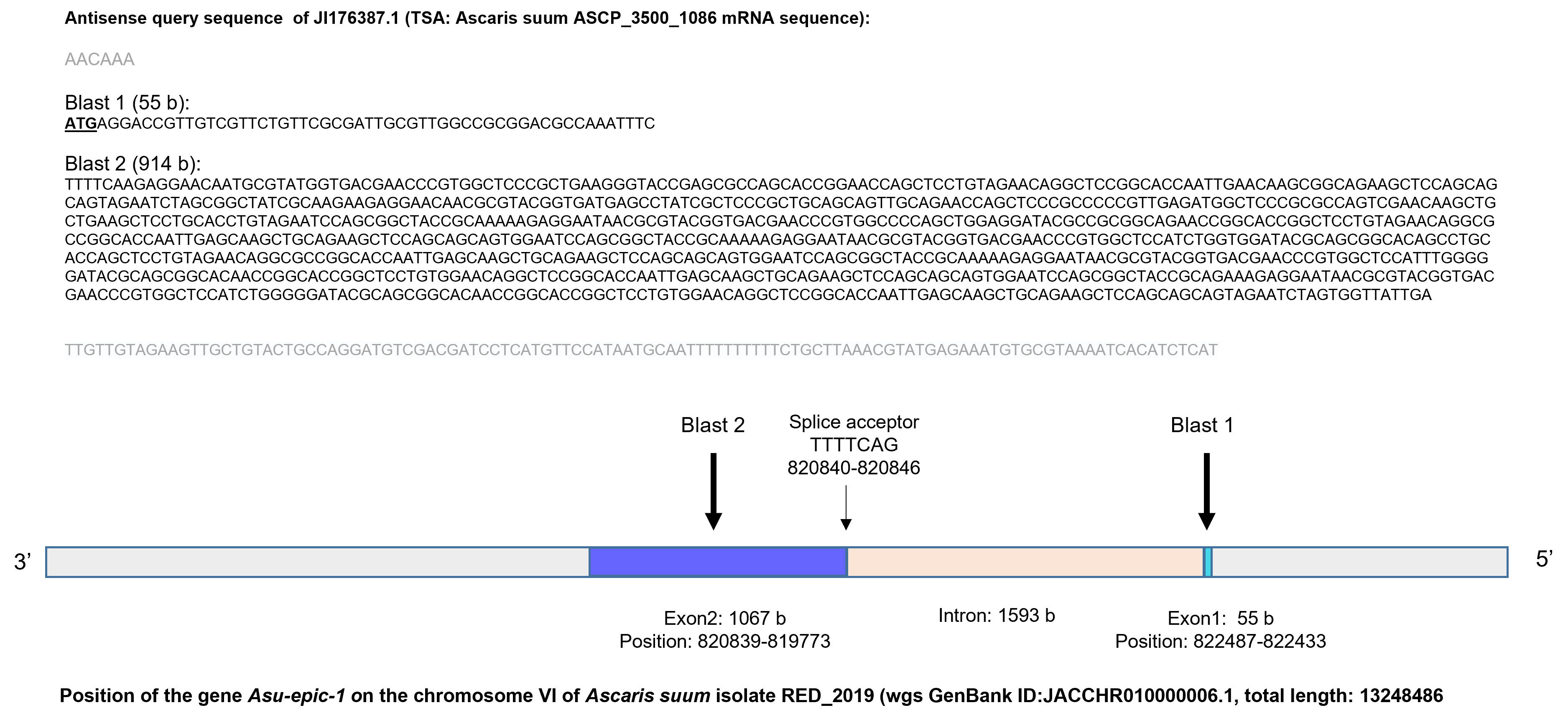


**S2 Figure.** Identification of the complete *Asu-epic-1* gene of *A. suum*. The antisense mRNA sequence JI176387 allowed the localization of the complete gene on the antisense strand of chromosome VI of the whole-genome shotgun contigs project (PRJNA62057 of isolate RED_2019). A blast with the 5’-coding region starting with the ATG initiation codon localized the exon1 (light blue) within the position 822487-822433. Another blast with the repeat-containing sequence of 914 bases localized the exon2 (dark blue) within positions 820839- 819773. A comparison of the gene and the mature RNA sequence confirms the involvement of a cis-splicing process which removes an intron of 1593 bases. Light grey nucleotides in JI176387 were excluded from the query sequence.
