# Supplementary material for: Identification and characterization of epicuticular proteins of nematodes sharing motifs with cuticular proteins of arthropods
Source: PLoS One. 2022 Oct 27;17(10):e0274751. doi: 10.1371/journal.pone.0274751 (PMC9612446; doi:10.1371/journal.pone.0274751)
Supplement: S3 Fig — (DOCX) [file pone.0274751.s003.docx]

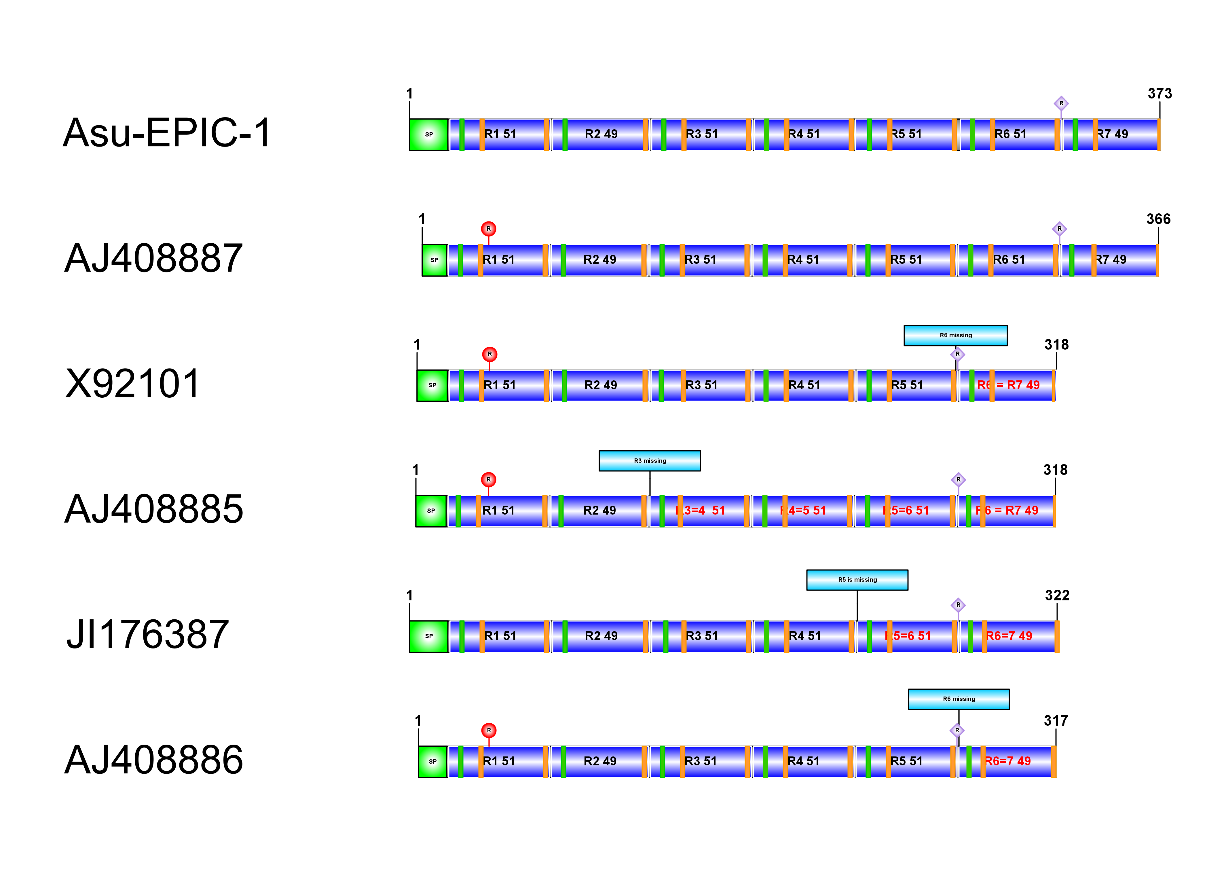


**S3 Figure.** Comparative alignment of the amino acid sequence of Asu-EPIC-1 with sequences encoded by four cDNA and one TSA clone (for further details, see Fig 3). Green boxes indicate the signal peptide. The repeats (blue boxes) are numbered, and the number of amino acids is given. The positions of the highly conserved motifs containing each one tyrosine are indicated as green (YGD) or orange (GYR) bars. Missing repeats are shown above the sequence position, and the repeats with red letters indicate the correct repeat number based on the unique nucleotide sequences. Amino acid variations are given as red circles. The rhombus indicates the amino acid substitution at the end of repeat 6 (R instead of K).
