# Supplementary material for: Identification and characterization of epicuticular proteins of nematodes sharing motifs with cuticular proteins of arthropods
Source: PLoS One. 2022 Oct 27;17(10):e0274751. doi: 10.1371/journal.pone.0274751 (PMC9612446; doi:10.1371/journal.pone.0274751)
Supplement: S1 Table — (DOCX) [file pone.0274751.s004.docx]

**S1 Table**. BLASTN results using the query AJ408887, an *A. suum* partial mRNA sequence coding for the first identified epicuticlin, in different databases*.

ENA database (em_all)

| Nr | Accession ID | Description | Length | S (Bit Score) | % Identity | Authors |
| --- | --- | --- | --- | --- | --- | --- |
| 1 | [EM_INV: AJ408887](https://www.ebi.ac.uk/ena/browser/view/AJ408887) | Ascaris suum partial mRNA for epicuticlin (epicut1 gene), clone C3. | 1298 | 2573.6 | 100 | Alaeddine 2001 |
| 2 | [EM_TSA: JI177333](https://www.ebi.ac.uk/ena/browser/view/JI177333) | TSA: Ascaris suum ASCP_3773_1017 mRNA sequence. | 1017 | 1939.2 | 99.1 | Wang et al. 2011 |
| 3 | [EM_INV: AJ408886](https://www.ebi.ac.uk/ena/browser/view/AJ408886) | Ascaris suum partial mRNA for epicuticlin (epicut1 gene), clone C2. | 1135 | 1788.6 | 99.1 | Alaeddine 2001 |
| 4 | [EM_INV: X92101](https://www.ebi.ac.uk/ena/browser/view/X92101) | Ascaris suum mRNA for cuticlin (epicut-1 gene). | 1597 | 1772.7 | 98.9 | Bisoffi 2001 |
| 5 | [EM_TSA: JI176387](https://www.ebi.ac.uk/ena/browser/view/JI176387) | TSA: Ascaris suum ASCP_3500_1086 mRNA sequence. | 1086 | 1693.4 | 97.9 | Wang et al. 2011 |
| 6 | [EM_TSA: JI178090](https://www.ebi.ac.uk/ena/browser/view/JI178090) | TSA: Ascaris suum ASCP_4016_966 mRNA sequence. | 966 | 1693.4 | 97.9 | Wang et al. 2011 |
| 7 | [EM_INV: AJ408885](https://www.ebi.ac.uk/ena/browser/view/AJ408885) | Ascaris suum partial mRNA for epicuticlin (epicut1 gene), clone C1. | 1053 | 1598.3 | 96.6 | Alaeddine 2001 |
| 8 | [EM_TSA: JI180250](https://www.ebi.ac.uk/ena/browser/view/JI180250) | TSA: Ascaris suum ASCP_4778_828 mRNA sequence. | 828 | 1586.4 | 99.2 | Wang et al. 2011 |
| 9 | [EM_EST: BM280956](https://www.ebi.ac.uk/ena/browser/view/BM280956) | ki07d07.y1 Ascaris suum L4 pSPORT1 Zarlenga v1 Ascaris suum cDNA 5' similar to TR: Q17090 Q17090 CUTICLIN ; mRNA sequence. | 633 | 1225.6 | 99.7 | McCarter et al. 2001 |
| 10 | [EM_EST: BM285155](https://www.ebi.ac.uk/ena/browser/view/BM285155) | kh95d02.y1 Ascaris suum L4 pSPORT1 Zarlenga v1 Ascaris suum cDNA 5' similar to TR: Q17090 Q17090 CUTICLIN ; mRNA sequence. | 626 | 1178 | 98.7 | McCarter et al. 2001 |
| 11 | [EM_EST: BM280996](https://www.ebi.ac.uk/ena/browser/view/BM280966) | ki07h10.y1 Ascaris suum L4 pSPORT1 Zarlenga v1 Ascaris suum cDNA 5' similar to TR: Q17090 Q17090 CUTICLIN ; mRNA sequence. | 615 | 1172.1 | 99.3 | McCarter et al. 2001 |
| 12 | [EM_EST: BM281293](https://www.ebi.ac.uk/ena/browser/view/BM281239) | ki11h11.y1 Ascaris suum L4 pSPORT1 Zarlenga v1 Ascaris suum cDNA 5' similar to TR: Q17090 Q17090 CUTICLIN ; mRNA sequence. | 627 | 1166.1 | 98.6 | McCarter et al. 2001 |
| 13 | [EM_EST: BM284807](https://www.ebi.ac.uk/ena/browser/view/BM284807) | kh99c10.y1 Ascaris suum L4 pSPORT1 Zarlenga v1 Ascaris suum cDNA 5' similar to TR: Q17090 Q17090 CUTICLIN ; mRNA sequence. | 572 | 1118.5 | 99.7 | McCarter et al. 2001 |
| 14 | [EM_EST: BM284867](https://www.ebi.ac.uk/ena/browser/view/BM284867) | kh92a05.y1 Ascaris suum L4 pSPORT1 Zarlenga v1 Ascaris suum cDNA 5' similar to TR: Q17090 Q17090 CUTICLIN ; mRNA sequence. | 582 | 1116.6 | 99.6 | McCarter et al. 2001 |
| 15 | [EM_EST: BM284673](https://www.ebi.ac.uk/ena/browser/view/BM284673) | kh97g05.y1 Ascaris suum L4 pSPORT1 Zarlenga v1 Ascaris suum cDNA 5' similar to TR: Q17090 Q17090 CUTICLIN ; mRNA sequence. | 582 | 1076.9 | 99.5 | McCarter et al. 2001 |
| 16 | [EM_EST: BM285248](https://www.ebi.ac.uk/ena/browser/view/BM285248) | kh96d11.y1 Ascaris suum L4 pSPORT1 Zarlenga v1 Ascaris suum cDNA 5' similar to TR: Q17090 Q17090 CUTICLIN ; mRNA sequence. | 554 | 1074.9 | 99.5 | McCarter et al. 2001 |
| 17 | [EM_EST: BI781495](https://www.ebi.ac.uk/ena/browser/view/BI781495) | kh12f11.y1 Ascaris suum female head pAMP1 v2 Chiapelli McCarter Ascaris suum cDNA 5' similar to TR: Q17090 Q17090 CUTICLIN ; mRNA sequence. | 549 | 1033.3 | 98.7 | McCarter et al. 2001 |
| 18 | [EM_EST: BI781775](https://www.ebi.ac.uk/ena/browser/view/BI781775) | kh01c03.y1 Ascaris suum female head pAMP1 v2 Chiapelli McCarter Ascaris suum cDNA 5' similar to TR: Q17090 Q17090 CUTICLIN ; mRNA sequence. | 543 | 999.6 | 99.4 | McCarter et al. 2001 |
| 19 | [EM_EST: BM281276](https://www.ebi.ac.uk/ena/browser/view/BM281276) | ki11g03.y1 Ascaris suum L4 pSPORT1 Zarlenga v1 Ascaris suum cDNA 5' similar to TR: Q17090 Q17090 CUTICLIN ; mRNA sequence. | 516 | 983.7 | 99 | McCarter et al. 2001 |
| 20 | [EM_EST: BI782671](https://www.ebi.ac.uk/ena/browser/view/BI782671) | kh28h01.y1 Ascaris suum male head pAMP1 v2 Chiapelli McCarter Ascaris suum cDNA 5' similar to TR: Q17090 Q17090 CUTICLIN ; mRNA sequence. | 515 | 946.1 | 99.6 | McCarter et al. 2001 |
| 21 | [EM_EST: BI594190](https://www.ebi.ac.uk/ena/browser/view/BI594190) | As_nc_07C05_SKPL Ascaris suum (parasitic nematode) adult nerve cord and muscle Ascaris suum cDNA clone As_nc_07C05 5' similar to emb\|CAC38984.1\| (AJ408889) hypothetical protein - Ascaris suum, mRNA sequence. | 611 | 942.1 | 96.2 | Blaxter et al.2001 |
| 22 | [EM_EST: BI781825](https://www.ebi.ac.uk/ena/browser/view/BI781825) | kh01h02.y1 Ascaris suum female head pAMP1 v2 Chiapelli McCarter Ascaris suum cDNA 5' similar to TR: Q17090 Q17090 CUTICLIN ; mRNA sequence. | 536 | 922.3 | 98 | McCarter et al. 2001 |
| 23 | [EM_EST: BI781225](https://www.ebi.ac.uk/ena/browser/view/BI781225) | kh09a12.y1 Ascaris suum female head pAMP1 v2 Chiapelli McCarter Ascaris suum cDNA 5' similar to TR: Q17090 Q17090 CUTICLIN ; mRNA sequence. | 452 | 888.6 | 99.8 | McCarter et al. 2001 |
| 24 | [EM_EST: BI594363](https://www.ebi.ac.uk/ena/browser/view/BI594363) | As_nc_11D06_SKPL Ascaris suum (parasitic nematode) adult nerve cord and muscle Ascaris suum cDNA clone As_nc_11D06 5' similar to emb\|CAC38984.1\| (AJ408889) hypothetical protein - Ascaris suum, mRNA sequence. | 458 | 829.1 | 97.8 | Blaxter et al.2001 |
| 25 | [EM_EST: BI782285](https://www.ebi.ac.uk/ena/browser/view/BI782285) | kh07g10.y1 Ascaris suum female head pAMP1 v2 Chiapelli McCarter Ascaris suum cDNA 5' similar to TR: Q17090 Q17090 CUTICLIN ; mRNA sequence. | 481 | 827.1 | 96.7 | McCarter et al. 2001 |
| 26 | [EM_EST: BI594244](https://www.ebi.ac.uk/ena/browser/view/BI594244) | As_nc_08G03_SKPL Ascaris suum (parasitic nematode) adult nerve cord and muscle Ascaris suum cDNA clone As_nc_08G03 5' similar to emb\|CAC38984.1\| (AJ408889) hypothetical protein - Ascaris suum, mRNA sequence. | 462 | 791.5 | 96.8 | Blaxter et al.2001 |
| 27 | [EM_EST: BG733615](https://www.ebi.ac.uk/ena/browser/view/BG733615) | As_bw_01E01_M13R Ascaris suum (parasitic nematode) body wall muscle and hypodermis Ascaris suum cDNA clone As_bw_01E01 5', mRNA sequence. | 429 | 632.9 | 98.5 | Blaxter et al.2001 |
| 28 | [EM_EST: BI781932](https://www.ebi.ac.uk/ena/browser/view/BI781932) | kh03c02.y1 Ascaris suum female head pAMP1 v2 Chiapelli McCarter Ascaris suum cDNA 5' similar to TR: Q17090 Q17090 CUTICLIN ; mRNA sequence. | 315 | 537.7 | 98.9 | McCarter et al. 2001 |
| 29 | [EM_EST: BI594697](https://www.ebi.ac.uk/ena/browser/view/BI594697) | As_nc_20D08_SKPL Ascaris suum (parasitic nematode) adult nerve cord and muscle Ascaris suum cDNA clone As_nc_20D08 5' similar to emb\|CAC38984.1\| (AJ408889) hypothetical protein - Ascaris suum, mRNA sequence. | 168 | 278 | 100 | Blaxter et al.2001 |
| 30 | [EM_EST: BG733705](https://www.ebi.ac.uk/ena/browser/view/BG733705) | As_nc_01B10_SKPL Ascaris suum (parasitic nematode) adult nerve cord and muscle Ascaris suum cDNA clone As_nc_01B10 5' similar to gb\|AAF66987.1\| | 691 | 77.8 | 100 | Blaxter et al.2001 |

EMBL-EBI DNA database

| Nr | Accession ID | Description | Length | S (Bit-Score) | % Identity | Authors |
| --- | --- | --- | --- | --- | --- | --- |
| 1 | [EM_INV: AJ408889](https://www.ebi.ac.uk/ena/data/view/AJ408889.1) | Ascaris suum epicut1 gene for epicuticlin and ORF1 DNA, clone G2 | 2022 | 3453.8 | 97 | Alaeddine 2001 |
| 2 | [EM_INV: AJ408890](https://www.ebi.ac.uk/ena/data/view/AJ408890.1) | Ascaris suum epicut1 gene for epicuticlin, clone G3 | 2130 | 3156.4 | 95.4 | Alaeddine 2001 |
| 3 | [EM_INV: AJ408888](https://www.ebi.ac.uk/ena/data/view/AJ408888.1) | Ascaris suum epicut1 gene for epicuticlin, clone G1 | 3590 | 2773.8 | 96.1 | Alaeddine 2001 |
| 4 | [EM_GSS: ED374165](https://www.ebi.ac.uk/ena/data/view/ED374165.1) | AUAC-aax00e09.b1 Ascaris suum whole genome shotgun library (PMAJ_4 GSS) Ascaris suum genomic, genomic survey sequence. | 833 | 1554.7 | 99.2 | Mitreva et al 2006 |
|  |  |  |  |  |  |  |
| NCBI WSG, database *A.suum* | | |  |  |  |  |
|  |  |  |  |  |  |  |
| Nr | Accession | Description | | S (Bit-Score) | % Identity | Authors |
| 1 | [JACCHR010000006](https://www.ncbi.nlm.nih.gov/nucleotide/JACCHR010000006.1?report=genbank&log$=nucltop&blast_rank=1&RID=2FU2MTN201R) | Ascaris suum isolate RED_2019 chromosome 6, whole genome shotgun sequence (PRJNA62057) | | 2233 | 99.2 | Wang et al 2020 |
| 2 | [ANBK02000042](https://www.ncbi.nlm.nih.gov/nuccore/ANBK02000042) | Ascaris suum isolate AS01 AsB12, whole genome shotgun sequence (PRJNA62057) | | 2217 | 98.9 | Wang et al 2017 |
| 3 | [AMPH01019725](https://www.ncbi.nlm.nih.gov/nuccore/AMPH01019725) | Ascaris suum Scaffold448_39, whole genome shotgun sequence (PRJNA80881) | | 1295 | 95.0 | Jex et al 2012 |
| 4 | [AEUI03000038](https://www.ncbi.nlm.nih.gov/nuccore/AEUI03000038) | Ascaris suum isolate AG01 AgB12, whole genome shotgun sequence (PRJNA62057) | | 699 | 99.5 | Wang et al 2017 |
| 5 | [AMPH01019726](https://www.ncbi.nlm.nih.gov/nuccore/AMPH01019726) | Ascaris suum Scaffold448_40, whole genome shotgun sequence (PRJNA80881) | | 595 | 97.1 | Jex et al 2012 |
| 6 | [AMPH01019727](https://www.ncbi.nlm.nih.gov/nuccore/AMPH01019727) | Ascaris suum Scaffold448_41, whole genome shotgun sequence (PRJNA80881) | | 62.1 | 100.0 | Jex et al 2012 |
|  |  |  |  |  |  |  |
| Wormbase ParaSite genome projects, database *A.suum* | | |  |  |  |  |
|  |  |  |  |  |  |  |
| Nr | Overlapping Genomic fragments | Genomic Location (Genome Project) | Orientation | Score (Bits) | % Identities | Authors |
| 1 | [GS_02605](https://parasite.wormbase.org/Ascaris_suum_prjna80881/Gene/Summary?db=core;g=GS_02605;tl=uVADN5hsYuFa4TxI-6142920-146008539) | Scaffold448:638294-638996 (PRJNA80881) | Reverse | 563 | 95 | Jex et al 2012 |
| 2 | [AgB12_g029](https://parasite.wormbase.org/Ascaris_suum_prjna62057/Gene/Summary?db=core;g=AgB12_g029;tl=uVADN5hsYuFa4TxI-6142920-146008491) | AgB12:666250-666633 (PRJNA62057 - AG01) | Forward | 376 | 99.5 | Wang et al 2011 |
| 3 | [AgB12_g029](https://parasite.wormbase.org/Ascaris_suum_prjna62057/Gene/Summary?db=core;g=AgB12_g029;tl=uVADN5hsYuFa4TxI-6142920-146008492) | AgB12:665602-665888 (PRJNA62057 - AG01) | Reverse | 263 | 97.9 | Wang et al 2011 |

* matches of *A.suum* sequences with identities equal or higher than 95% are listed and sorted according to decreasing Scores. % Identity indicates the % of nucleotide similarity in the alignment.
