# Supplementary material for: Identification and characterization of epicuticular proteins of nematodes sharing motifs with cuticular proteins of arthropods
Source: PLoS One. 2022 Oct 27;17(10):e0274751. doi: 10.1371/journal.pone.0274751 (PMC9612446; doi:10.1371/journal.pone.0274751)
Supplement: S2 Table — (DOCX) [file pone.0274751.s005.docx]

**S2 Table.** Epicuticlin sequences detected in different nematode species^1^.

Clade III Spirurina

| **N Species** | **Species Name** | **N proteins** | **Uniprot** | **NCBI** | **Locus Tag** | **Protein name^2^** | **N of aa** | **N of repeats** |
| --- | --- | --- | --- | --- | --- | --- | --- | --- |
| 1 | *Acanthocheilonema viteae* | 1 | [A0A498SKB1 (ACAVI)](https://www.uniprot.org/uniprot/A0A498SKB1) | [VBB32276](https://www.ncbi.nlm.nih.gov/protein/VBB32276?report=genbank&log$=taxrep&RID=5MZ5BRS0014) | NAV_LOCUS7067 | Avi-EPIC-x | 55 | 1 |
| 2 | *Anisakis simplex* | 2 | [A0A0M3K446 (ANISI)](https://www.uniprot.org/uniprot/A0A0M3K446) | [VDK54374](https://www.ncbi.nlm.nih.gov/protein/VDK54374?report=genbank&log$=taxrep&RID=6Z06XPZJ016) | ASIM_LOCUS15145 | Asi-EPIC-x | 107 | 2 |
| 3 | *Ascaris lumbricoides* | 3 | [A0A0M3I7G7 (ASCLU)](https://www.uniprot.org/uniprot/A0A0M3I7G7) | n/a | ALUE_0001312801 | Alu-EPIC-1 | 344 | 6 |
|  |  | 4 | [A0A0M3I7G8 (ASCLU)](https://www.uniprot.org/uniprot/A0A0M3I7G8) | n/a | ALUE_0001312901 | Alu-EPIC-2 | 389 | 6 |
|  |  | 5 | [A0A0M3IF25 (ASCLU)](https://www.uniprot.org/uniprot/A0A0M3IF25) | n/a | ALUE_0001677101 | Alu-EPIC-3 | 107 | 2 |
| 4 | *Ascaris suum* | 6 | n/a | TPA: BK061342 | n/a | Asu-EPIC-1 | 373 | 7 |
|  |  | 7 | n/a | n/a | n/a | Asu-EPIC-2 | 419 | 8 |
|  |  | 8 | n/a | [AgB12_g028_t01](https://parasite.wormbase.org/Ascaris_suum_prjna62057/Transcript/Sequence_Protein?g=AgB12_g028;r=AgB12:654298-657498;t=AgB12_g028_t01) (Wormbase parasite) | n/a | Asu-EPIC-3 | 405 | 5 |
| 5 | *Brugia malayi* | 9 | [A0A4E9FS76 (BRUMA)](https://www.uniprot.org/uniprot/A0A4E9FS76) | [VIO98620](https://www.ncbi.nlm.nih.gov/protein/VIO98620?report=genbank&log$=taxrep&RID=6Z06XPZJ016) | Bm1_51485 | Bma-EPIC-x | 187 | 3 |
|  |  | 10 | [A0A0H5S6L0 (BRUMA)](https://www.uniprot.org/uniprot/A0A0H5S6L0) | [CRZ24377](https://www.ncbi.nlm.nih.gov/protein/CRZ24377.1) | Bm4912 | Bma-EPIC-x | 252 | 3 |
| 6 | *Brugia pahangi* | 11 | [A0A0N4T004 (BRUPA)](https://www.uniprot.org/uniprot/A0A0N4T004) | [VDN82586](https://www.ncbi.nlm.nih.gov/protein/VDN82586?report=genbank&log$=taxrep&RID=6Z06XPZJ016) | BPAG_LOCUS1400 | Bpa-EPIC-x | 261 | 3 |
| 7 | *Brugia timori* | 12 | [A0A0R3QDI6 (9BILA)](https://www.uniprot.org/uniprot/A0A0R3QDI6) | [VDO15370](https://www.ncbi.nlm.nih.gov/protein/VDO15370?report=genbank&log$=taxrep&RID=5MZ5BRS0014) | BTMF_LOCUS3719 | Bti-EPIC-x | 81 | 2 |
| 8 | *Dracunculus medinensis* | 13 | [A0A0N4US81 (DRAME)](https://www.uniprot.org/uniprot/A0A0N4US81) | [VDN54331](https://www.ncbi.nlm.nih.gov/protein/VDN54331?report=genbank&log$=taxrep&RID=6Z06XPZJ016) | DME_LOCUS4304 | Dme-EPIC-x | 163 | 3 |
|  |  | 14 | [A0A0N4UJ46 (DRAME)](https://www.uniprot.org/uniprot/A0A0N4UJ46) | [VDN60121](https://www.ncbi.nlm.nih.gov/protein/VDN60121?report=genbank&log$=taxrep&RID=5MZ5BRS0014) | DME_LOCUS10094 | Dme-EPIC-x | 124 | 2 |
|  |  | 15 | [A0A0N4UMW3 (DRAME)](https://www.uniprot.org/uniprot/A0A0N4UMW3) | [VDN52978](https://www.ncbi.nlm.nih.gov/protein/VDN52978?report=genbank&log$=taxrep&RID=5MZ5BRS0014) | DME_LOCUS2951 | Dme-EPIC-y | 121 | 2 |
| 9 | *Gongylonema pulchrum* | 16 | [A0A183CU93 (9BILA)](https://www.uniprot.org/uniprot/A0A183CU93) | [VDK27129](https://www.ncbi.nlm.nih.gov/protein/VDK27129?report=genbank&log$=taxrep&RID=6Z06XPZJ016) | GPUH_LOCUS34 | Gpu-EPIC-x | 303 | 6 |
|  |  | 17 | [A0A183CYK0 (9BILA)](https://www.uniprot.org/uniprot/A0A183CYK0) | [VDK30335](https://www.ncbi.nlm.nih.gov/protein/VDK30335?report=genbank&log$=taxrep&RID=6Z06XPZJ016) | PUH_LOCUS1541 | Gpu-EPIC-y | 170 | 3 |
| 10 | *Litomosoides sigmodontis* | 18 | [A0A3P6UBA8 (LITSI)](https://www.uniprot.org/uniprot/A0A3P6UBA8) | [VDK74351](https://www.ncbi.nlm.nih.gov/protein/VDK74351?report=genbank&log$=taxrep&RID=5MZ5BRS0014) | NLS_LOCUS2441 | Lis-EPIC-x | 136 | 2 |
| 11 | *Loa loa* | 19 | [A0A1I7VRZ5 (LOALO)](https://www.uniprot.org/uniprot/A0A1I7VRZ5) | [XP_020302978](https://www.ncbi.nlm.nih.gov/protein/XP_020302978?report=genbank&log$=taxrep&RID=5MZ5BRS0014) | EN70_563 | Llo-EPIC-x | 272 | 5 |
|  |  | 20 | [A0A1S0U1W3 (LOALO)](https://www.uniprot.org/uniprot/A0A1S0U1W3) | [EFO23565](https://www.ncbi.nlm.nih.gov/protein/EFO23565?report=genbank&log$=taxrep&RID=5MZ5BRS0014) | LOAG_04915 | Llo-EPIC-x | 164 | 3 |
| 12 | *Onchocerca flexuosa* | 21 | [A0A183H955 (9BILA)](https://www.uniprot.org/uniprot/A0A183H955) | [OZC07268](https://www.ncbi.nlm.nih.gov/protein/OZC07268?report=genbank&log$=taxrep&RID=5MZ5BRS0014) | X798_05747 | Ofo-EPIC-x | 94 | 1 |
|  |  | 22 | [A0A238BLU2 (9BILA)](https://www.uniprot.org/uniprot/A0A238BLU2) | [OZC06371](https://www.ncbi.nlm.nih.gov/protein/OZC06371?report=genbank&log$=taxrep&RID=5MZ5BRS0014) | X798_06638 | Ofo-EPIC-y | 111 | 1 |
|  |  | 23 | [A0A238BRR0 (9BILA)](https://www.uniprot.org/uniprot/A0A238BRR0) | [VDO38603](https://www.ncbi.nlm.nih.gov/protein/VDO38603?report=genbank&log$=taxrep&RID=5MZ5BRS0014) | OFLC_LOCUS4019 | Ofo-EPIC-z | 111 | 1 |
| 13 | *Onchocerca ochengi* | 24 | [A0A182ERS1 (ONCOC)](https://www.uniprot.org/uniprot/A0A182ERS1) | [VDK75900](https://www.ncbi.nlm.nih.gov/protein/VDK75900?report=genbank&log$=taxrep&RID=5MZ5BRS0014) | NOO_LOCUS5075 | Ooc-EPIC-x | 92 | 1 |
|  |  | 25 | [A0A182EAJ9 (ONCOC)](https://www.uniprot.org/uniprot/A0A182EAJ9) | [VDM94566](https://www.ncbi.nlm.nih.gov/protein/VDM94566?report=genbank&log$=taxrep&RID=5MZ5BRS0014) | NOO_LOCUS10839 | Ooc-EPIC-x | 104 | 1 |
| 14 | *Onchocerca volvulus* | 26 | [A0A044RSS6 (ONCVO)](https://www.uniprot.org/uniprot/A0A044RSS6) | n/a | WBGene00249462 | Ovo-EPIC-x | 116 | 1 |
|  |  | 27 | [A0A044UFI9 (ONCVO)](https://www.uniprot.org/uniprot/A0A044UFI9) | n/a | [WBGene00243705](http://www.ensemblgenomes.org/id/WBGene00243705) | Ovo-EPIC-x | 111 | 1 |
| 15 | *Thelazia callipaeda* | 28 | [A0A0N5CRU2 (THECL)](https://www.uniprot.org/uniprot/A0A0N5CRU2) | [VDM99163](https://www.ncbi.nlm.nih.gov/protein/VDM99163?report=genbank&log$=taxrep&RID=5MZ5BRS0014) | TCLT_LOCUS2943 | Thc-EPIC-x | 82 | 2 |
| 16 | *Toxocara canis* | 29 | [A0A183TVS5 (TOXCA)](https://www.uniprot.org/uniprot/A0A183TVS5) | [VDM24067](https://www.ncbi.nlm.nih.gov/protein/VDM24067?report=genbank&log$=taxrep&RID=6Z06XPZJ016) | TCNE_LOCUS345 | Tca-EPIC-1 | 469 | 9 |
|  |  | 30 | [A0A0B2UZ77 (TOXCA)](https://www.uniprot.org/uniprot/A0A0B2UZ77) | [KHN74524](https://www.ncbi.nlm.nih.gov/protein/KHN74524?report=genbank&log$=taxrep&RID=6Z06XPZJ016) | Tcan_16096 | Tca-EPIC-1 | 1810 | 32 |
|  |  | 31 | [A0A183V326 (TOXCA)](https://www.uniprot.org/uniprot/A0A183V326) | [VDM46467](https://www.ncbi.nlm.nih.gov/protein/VDM46467?report=genbank&log$=taxrep&RID=6Z06XPZJ016) | TCNE_LOCUS15146 | Tca-EPIC-2 | 165 | 3 |
|  |  | 32 | [A0A183TVS6 (TOXCA)](https://www.uniprot.org/uniprot/A0A183TVS6) | [VDM24068](https://www.ncbi.nlm.nih.gov/protein/VDM24068?report=genbank&log$=taxrep&RID=6Z06XPZJ016) | TCNE_LOCUS346 | Tca-EPIC-3 | 288 | 3 |
|  |  | 33 | [A0A0B2V0B7 (TOXCA)](https://www.uniprot.org/uniprot/A0A0B2V0B7) | [KHN74525](https://www.ncbi.nlm.nih.gov/protein/KHN74525?report=genbank&log$=taxrep&RID=6Z06XPZJ016) | Tcan_16099 | Tca-EPIC-3 | 1969 | 33 |
| 17 | *Wuchereria bancrofti* | 34 | [A0A3P7FAS6 (WUCBA)](https://www.uniprot.org/uniprot/A0A3P7FAS6) | [EJW88123](https://www.ncbi.nlm.nih.gov/protein/EJW88123?report=genbank&log$=taxrep&RID=5MZ5BRS0014) | WUBG_00963 | Wba-EPIC-x | 98 | 2 |
|  |  | 35 | [J9F0W6 (WUCBA)](https://www.uniprot.org/uniprot/J9F0W6) | [VDM07652](https://www.ncbi.nlm.nih.gov/protein/VDM07652?report=genbank&log$=taxrep&RID=5MZ5BRS0014) | WBA_LOCUS1038 | Wba-EPIC-x | 114 | 3 |
|  |  | 36 | [A0A1I8EE92 (WUCBA)](https://www.uniprot.org/uniprot/A0A1I8EE92) | n/a | maker-PairedContig 1697-snap-gene-1.13 | Wba-EPIC-x | 157 | 3 |

Clade IV Tylenchina

| **N Species** | **Species Name** | **N proteins** | **Uniprot** | **NCBI** | **Locus Tag** | **Protein name^2^** | **N of aa** | **N of repeats** |
| --- | --- | --- | --- | --- | --- | --- | --- | --- |
| 18 | *Bursaphelenchus xylophilus* | 37 | [A0A1I7RHX4 (BURXY)](https://www.uniprot.org/uniprot/A0A1I7RHX4) | n/a | BXY_0030300 | Bxy-EPIC-x | 380 | 7 |
|  |  | 38 | [A0A1I7RXY2 (BURXY)](https://www.uniprot.org/uniprot/A0A1I7RXY2) | n/a | BXY_0559900 | Bxy-EPIC-y | 114 | 1 |
| 19 | *Halicephalobus sp. NKZ332* | 39 | [A0A6G0V1Q0_9BILA](https://www.uniprot.org/uniprot/A0A6G0V1Q0) | [KAE9554809](https://www.ncbi.nlm.nih.gov/protein/KAE9554809?report=genbank&log$=taxrep&RID=5MZ5BRS0014) | FO519_002006 | Hsp-EPIC-x | 215 | 4 |
|  |  | 40 | [A0A6G0V5L8_9BILA](https://www.uniprot.org/uniprot/A0A6G0V5L8) | [KAE9555726](https://www.ncbi.nlm.nih.gov/protein/KAE9555726?report=genbank&log$=taxrep&RID=5MZ5BRS0014) | FO519_001078 | Hsp-EPIC-y | 198 | 2 |
| 20 | *Meloidogyne hapla* | 41 | [A0A1I8B235 (MELHA)](https://www.uniprot.org/uniprot/A0A1I8B235) | n/a | MhA1_Contig125.frz3.gene5 | Mha-EPIC-x | 378 | 4 |
| 21 | *Parastrongyloides trichosuri* | 42 | [A0A0N5A1S0 (PARTI)](https://www.uniprot.org/uniprot/A0A0N5A1S0) | n/a | PTRK_0001556900 | Ptr-EPIC-x | 156 | 2 |
| 22 | *Rhabditophanes sp.* | 43 | [A0A1I8CET0 (9BILA)](https://www.uniprot.org/uniprot/A0A1I8CET0) | n/a | RSKR_0000340400 | Rsp-EPIC-x | 277 | 3 |
| 23 | *Steinernema carpocapsae* | 44 | [A0A4U8V0N4 (STECR)](https://www.uniprot.org/uniprot/A0A4U8V0N4) | n/a | L596_005459 | Sca-EPIC-x | 184 | 3 |
| 24 | *Steinernema glaseri* | 45 | [A0A1I7YLQ6 (9BILA)](https://www.uniprot.org/uniprot/A0A1I7YLQ6) | n/a | L893_g17588 | Sgl-EPIC-x | 342 | 6 |
|  |  | 46 | [A0A1I8ANA2 (9BILA)](https://www.uniprot.org/uniprot/A0A1I8ANA2) | n/a | L893_g7194 | Sgl-EPIC-y | 342 | 6 |
|  |  | 47 | [A0A1I8AUW5 (9BILA)](https://www.uniprot.org/uniprot/A0A1I8AUW5) | n/a | L893_g9426 | Sgl-EPIC-z | 452 | 2 |
| 25 | *Strongyloides papillosus* | 48 | [A0A0N5C866 (STREA)](https://www.uniprot.org/uniprot/A0A0N5C866) | n/a | SPAL_0001412100 | Spa-EPIC-x | 155 | 2 |
| 26 | *Strongyloides ratti* | 49 | [A0A090LFQ4 (STRRB)](https://www.uniprot.org/uniprot/A0A090LFQ4) | [XP_024507792](https://www.ncbi.nlm.nih.gov/protein/XP_024507792?report=genbank&log$=taxrep&RID=6Z06XPZJ016) | SRAE_2000324800 | Sra-EPIC-x | 355 | 5 |
|  |  | 50 | [A0A090LA87 (STRRB)](https://www.uniprot.org/uniprot/A0A090LA87) | [XP_024505847](https://www.ncbi.nlm.nih.gov/protein/XP_024505847?report=genbank&log$=taxrep&RID=5MZ5BRS0014) | SRAE_2000131500 | Sra-EPIC-y | 132 | 2 |
| 27 | *Strongyloides stercoralis* | 51 | [A0A0K0DTE3 (STRER)](https://www.uniprot.org/uniprot/A0A0K0DTE3) | n/a | SSTP_0000050500 | Sst-EPIC-x | 278 | 4 |
|  |  | 52 | [A0A0K0EF07 (STRER)](https://www.uniprot.org/uniprot/A0A0K0EF07) | n/a | SSTP_0000807100 | Sst-EPIC-y | 135 | 2 |
| 28 | *Strongyloides venezuelensis* | 53 | [A0A0K0FXV1 (STRVS)](https://www.uniprot.org/uniprot/A0A0K0FXV1) | n/a | [SVE_1727700](https://parasite.wormbase.org/id/SVE_1727700) | Sve-EPIC-x | 138 | 2 |
|  |  | 54 | [A0A0K0G246 (STRVS)](https://www.uniprot.org/uniprot/A0A0K0G246) | n/a | [SVE_1879200](https://parasite.wormbase.org/id/SVE_1879200) | Sve-EPIC-y | 134 | 2 |
| 29 | *Strongylus vulgaris* | 55 | [A0A3P7LJ15 (STRVU)](https://www.uniprot.org/uniprot/A0A3P7LJ15) | [VDM82575](https://www.ncbi.nlm.nih.gov/protein/VDM82575?report=genbank&log$=taxrep&RID=5MZ5BRS0014) | SVUK_LOCUS17573 | Svu-EPIC-x | 159 | 2 |
|  |  | 56 | [A0A3P7KD62_STRVU](https://www.uniprot.org/uniprot/A0A3P7KD62) | [VDM69195](https://www.ncbi.nlm.nih.gov/protein/VDM69195?report=genbank&log$=taxrep&RID=5MZ5BRS0014) | SVUK_LOCUS4193 | Svu-EPIC-y | 141 | 2 |
|  |  |  |  |  |  |  |  |  |
| Clade V Rhabditina | |  |  |  |  |  |  |  |
| **N Species** | **Species Name** | **N proteins** | **Uniprot** | **NCBI** | **Locus Tag** | **Protein name^2^** | **N of aa^3^** | **N of repeats** |
| 30 | *Ancylostoma caninum* | 57 | [A0A368GWQ4 (ANCCA)](https://www.uniprot.org/uniprot/A0A368GWQ4) | [RCN48782](https://www.ncbi.nlm.nih.gov/protein/RCN48782?report=genbank&log$=taxrep&RID=5MZ5BRS0014) | ANCCAN_05065 | Aca-EPIC-x | **501** | 9 |
|  |  | 58 | [A0A368GWR3 (ANCCA)](https://www.uniprot.org/uniprot/A0A368GWR3) | [RCN48781](https://www.ncbi.nlm.nih.gov/protein/RCN48781?report=genbank&log$=taxrep&RID=5MZ5BRS0014) | ANCCAN_05064 | Aca-EPIC-y | 326 | 4 |
| 31 | *Ancylostoma ceylanicum* | 59 | [A0A016WQ83 (9BILA)](https://www.uniprot.org/uniprot/A0A016WQ83) | [EYC41956](https://www.ncbi.nlm.nih.gov/protein/EYC41956?report=genbank&log$=taxrep&RID=6Z06XPZJ016) | Acey_s0550.g3316 | Ace-EPIC-x | **338** | 5 |
|  |  | 60 | [A0A016WQS2 (9BILA)](https://www.uniprot.org/uniprot/A0A016WQS2) | [EYC41959](https://www.ncbi.nlm.nih.gov/protein/EYC41959?report=genbank&log$=taxrep&RID=6Z06XPZJ016) | Acey_s0550.g3313 | Ace-EPIC-y | 541 | 6 |
|  |  | 61 | A0A016WZ87_9BILA | [EYC44348](https://www.ncbi.nlm.nih.gov/protein/EYC44348?report=genbank&log$=taxrep&RID=5MZ5BRS0014) | Acey_s0464.g1933 | Ace-EPIC-z | 139 | 1 |
| 32 | *Ancylostoma duodenale* | 62 | [A0A0C2CUJ4 (9BILA)](https://www.uniprot.org/uniprot/A0A0C2CUJ4) | [KIH53487](https://www.ncbi.nlm.nih.gov/protein/KIH53487?report=genbank&log$=taxrep&RID=6Z06XPZJ016) | ANCDUO_16385 | Adu-EPIC-x | **224** | 4 |
| 33 | *Angiostrongylus cantonensis* | 63 | n/a | [KAE9417512](https://www.ncbi.nlm.nih.gov/protein/KAE9417512?report=genbank&log$=taxrep&RID=5MZ5BRS0014) | Angca_001007 | Anca-EPIC-x | **101** | 1 |
| 34 | *Angiostrongylus costaricensis* | 64 | [A0A0R3PW20_ANGCS](https://www.uniprot.org/uniprot/A0A0R3PW20) | [VDM61879](https://www.ncbi.nlm.nih.gov/protein/VDM61878?report=genbank&log$=taxrep&RID=5MZ5BRS0014) | ACOC_LOCUS10294 | Anco-EPIC-x | **177** | 3 |
| 35 | *Caenorhabditis brenneri* | 65 | [G0MRF0 (CAEBE)](https://www.uniprot.org/uniprot/G0MRF0) | [EGT42147](https://www.ncbi.nlm.nih.gov/protein/EGT42147?report=genbank&log$=taxrep&RID=6Z06XPZJ016) | CAEBREN_23336 | Cbn-EPIC-y | 347 | 6 |
|  |  | 66 | [G0N6L1 (CAEBE)](https://www.uniprot.org/uniprot/G0N6L1) | [EGT53856](https://www.ncbi.nlm.nih.gov/protein/EGT53856?report=genbank&log$=taxrep&RID=6Z06XPZJ016) | CAEBREN_08277 | Cbn-EPIC-x | **636** | 10 |
| 36 | *Caenorhabditis briggsae* | 67 | [A8WN32 (CAEBR)](https://www.uniprot.org/uniprot/A8WN32) | [XP_002632411](https://www.ncbi.nlm.nih.gov/protein/XP_002632411?report=genbank&log$=taxrep&RID=6Z06XPZJ016) | CBG00436 | Cbr-EPIC-y | 348 | 6 |
|  |  | 68 | [A8XI93 (CAEBR)](https://www.uniprot.org/uniprot/A8XI93) | [CAP21887](https://www.ncbi.nlm.nih.gov/protein/CAP21887?report=genbank&log$=taxrep&RID=6Z06XPZJ016) | CBG13589 | Cbr-EPIC-x | **452** | 7 |
|  |  | 69 | [A8X1T0 (CAEBR)](https://www.uniprot.org/uniprot/A8X1T0) | [XP_002635043](https://www.ncbi.nlm.nih.gov/protein/XP_002635043?report=genbank&log$=taxrep&RID=6Z06XPZJ016) | CBG05723 | Cbr-EPIC-z | 120 | 1 |
| 37 | *Caenorhabditis elegans* | 70 | [Q9U3J8 (CAEEL)](https://www.uniprot.org/uniprot/Q9U3J8) | [NP_503116 /CAB62800](https://www.ncbi.nlm.nih.gov/protein/NP_503116?report=genbank&log$=taxrep&RID=6Z06XPZJ016) | CELE_F11E6.3 | Cel-EPIC-y | 345 | 6 |
|  |  | 71 | [Q8MXU8 (CAEEL)](https://www.uniprot.org/uniprot/Q8MXU8) | [NP_741325 /CCD72804](https://www.ncbi.nlm.nih.gov/protein/NP_741325?report=genbank&log$=taxrep&RID=6Z06XPZJ016) | CELE_K08D12.6 | Cel-EPIC-x | **668** | 10 |
| 38 | *Caenorhabditis japonicum* | 72 | [A0A2H2I3A1 (CAEJA)](https://www.uniprot.org/uniprot/A0A2H2I3A1) | n/a | WBGene00125230 | Cjp-EPIC-y | 495 | 9 |
|  |  | 73 | [A0A2H2IQ27 (CAEJA)](https://www.uniprot.org/uniprot/A0A2H2IQ27) | n/a | [WBGene00176164](http://www.ensemblgenomes.org/id/WBGene00176164) | Cjp-EPIC-x | **386** | 6 |
| 39 | *Caenorhabditis latens* | 74 | [A0A261BUD9 (9PELO)](https://www.uniprot.org/uniprot/A0A261BUD9) | [OZG13929](https://www.ncbi.nlm.nih.gov/protein/OZG13929?report=genbank&log$=taxrep&RID=6Z06XPZJ016) | FL83_12136 | Cla-EPIC-x | **637** | 10 |
|  |  | 75 | [A0A260ZP79 (9PELO)](https://www.uniprot.org/uniprot/A0A260ZP79) | [OZF87473](https://www.ncbi.nlm.nih.gov/protein/OZF87473?report=genbank&log$=taxrep&RID=6Z06XPZJ016) | FL83_22919 /FL83_22901 | Cla-EPIC-y | 355 | 6 |
| 40 | *Caenorhabditis nigoni* | 76 | [A0A2G5TVA2 (9PELO)](https://www.uniprot.org/uniprot/A0A2G5TVA2) | [PIC31198](https://www.ncbi.nlm.nih.gov/protein/PIC31198?report=genbank&log$=taxrep&RID=6Z06XPZJ016) | Cni-F11E6.3 | Cni-EPIC-y | 399 | 7 |
|  |  | 77 | [A0A2G5UDR8 (9PELO)](https://www.uniprot.org/uniprot/A0A2G5UDR8) | [PIC37613](https://www.ncbi.nlm.nih.gov/protein/PIC37613?report=genbank&log$=taxrep&RID=6Z06XPZJ016) | Cni-K08D12.6 | Cni-EPIC-x | **514** | 8 |
| 41 | *Caenorhabditis remanei* | 78 | [A0A261A3I2 (CAERE)](https://www.uniprot.org/uniprot/A0A261A3I2) | [OZF92075](https://www.ncbi.nlm.nih.gov/protein/OZF92075?report=genbank&log$=taxrep&RID=6Z06XPZJ016) | FL82_20653 | Cre-EPIC-y | 470 | 6 |
|  |  | 79 | [E3NJW7 (CAERE)](https://www.uniprot.org/uniprot/E3NJW7) | [OZG01079](https://www.ncbi.nlm.nih.gov/protein/OZG01079?report=genbank&log$=taxrep&RID=6Z06XPZJ016) | CRE_23773 | Cre-EPIC-x | **642** | 10 |
| 42 | *Caenorhabditis tropicalis* | 80 | [A0A1I7V4N7 (9PELO)](https://www.uniprot.org/uniprot/A0A1I7V4N7) | n/a | Csp11.Scaffold70.g423 | Ctr-EPIC-y | 344 | 6 |
|  |  | 81 | [A0A1I7TPV0 (9PELO)](https://www.uniprot.org/uniprot/A0A1I7TPV0) | n/a | Csp11.Scaffold629.g10586 | Ctr-EPIC-x | **151** | 2 |
| 43 | *Cylicostephanus goldi* | 82 | [A0A3P6TUB3 (CYLGO)](https://www.uniprot.org/uniprot/A0A3P6TUB3) | [VDK82680](https://www.ncbi.nlm.nih.gov/protein/VDK82680?report=genbank&log$=taxrep&RID=5MZ5BRS0014) | CGOC_LOCUS8013 | Cgo-EPIC-y | 91 | 1 |
| 44 | *Diploscapter pachys* | 83 | [A0A2A2KZD1 (9BILA)](https://www.uniprot.org/uniprot/A0A2A2KZD1) | [PAV79242](https://www.ncbi.nlm.nih.gov/protein/PAV79242?report=genbank&log$=taxrep&RID=6Z06XPZJ016) | WR25_03827 | Dpa-EPIC-y | 254 | 4 |
|  |  | 84 | [A0A2A2JRP1 (9BILA)](https://www.uniprot.org/uniprot/A0A2A2JRP1) | [PAV57329](https://www.ncbi.nlm.nih.gov/protein/PAV57329?report=genbank&log$=taxrep&RID=5MZ5BRS0014) | WR25_13729 | Dpa-EPIC-x | **279** | 4 |
| 45 | *Elaeophora elaphi* | 85 | [A0A0R3RUZ9 (9BILA)](https://www.uniprot.org/uniprot/A0A0R3RUZ9) | n/a | [EEL_0000588601](https://parasite.wormbase.org/id/EEL_0000588601) | Eel-EPIC-y | 55 | 1 |
| 46 | *Haemonchus contortus* | 86 | n/a | [CDJ81321](https://www.ncbi.nlm.nih.gov/protein/CDJ81321?report=genbank&log$=taxrep&RID=6Z06XPZJ016) | HCOI_00291400 | Hco-EPIC-y | 577 | 5 |
|  |  | 87 | n/a | [CDJ81322](https://www.ncbi.nlm.nih.gov/protein/CDJ81322?report=genbank&log$=taxrep&RID=6Z06XPZJ016) | HCOI_00291500 | Hco-EPIC-x | **472** | 8 |
| 47 | *Haemonchus placei* | 88 | [A0A0N4WHH1 (HAEPC)](https://www.uniprot.org/uniprot/A0A0N4WHH1) | [VDO39818](https://www.ncbi.nlm.nih.gov/protein/VDO39818?report=genbank&log$=taxrep&RID=6Z06XPZJ016) | HPLM_LOCUS10323 | Hpl-EPIC-y | 329 | 5 |
|  |  | 89 | [A0A0N4WHH0 (HAEPC)](https://www.uniprot.org/uniprot/A0A0N4WHH0) | [VDO39816](https://www.ncbi.nlm.nih.gov/protein/VDO39816?report=genbank&log$=taxrep&RID=6Z06XPZJ016) | HPLM_LOCUS10322 | Hpl-EPIC-x | **290** | 5 |
|  |  | 90 | [A0A0N4VZT0 (HAEPC)](https://www.uniprot.org/uniprot/A0A0N4VZT0) | [VDO16906](https://www.ncbi.nlm.nih.gov/protein/VDO16906?report=genbank&log$=taxrep&RID=5MZ5BRS0014) | HPLM_LOCUS2798 | Hpl-EPIC-z | 129 | 1 |
| 48 | *Heligmosomoides polygyrus* | 91 | [A0A183G796 (HELPZ)](https://www.uniprot.org/uniprot/A0A183G796) | [VDP09482](https://www.ncbi.nlm.nih.gov/protein/VDP09482?report=genbank&log$=taxrep&RID=6Z06XPZJ016) | HPBE_LOCUS17639 | Hpo-EPIC-x | **292** | 5 |
|  |  | 92 | [A0A183G797 (HELPZ)](https://www.uniprot.org/uniprot/A0A183G797) | [VDP09486](https://www.ncbi.nlm.nih.gov/protein/VDP09486?report=genbank&log$=taxrep&RID=5MZ5BRS0014) | HPBE_LOCUS17640 | Hpo-EPIC-z | 307 | 2 |
| 49 | *Heterorhabditis bacteriophora* | 93 | [A0A1I7XR61 (HETBA)](https://www.uniprot.org/uniprot/A0A1I7XR61) | n/a | Hba_20219 | Hba-EPIC-y | 404 | 4 |
|  |  | 94 | [A0A1I7XQZ4_HETBA](https://www.uniprot.org/uniprot/A0A1I7XQZ4) | n/a | Hba_20220 | Hba-EPIC-z | 128 | 2 |
| 50 | *Necator americanus* | 95 | [W2T817 (NECAM)](https://www.uniprot.org/uniprot/W2T817) | [XP_013300392](https://www.ncbi.nlm.nih.gov/protein/XP_013300392?report=genbank&log$=taxrep&RID=5MZ5BRS0014) | NECAME_18243 | Nam-EPIC-x | **99** | 1 |
|  |  | 96 | [W2SKT7_NECAM](https://www.uniprot.org/uniprot/W2SKT7) | [ETN69481](https://www.ncbi.nlm.nih.gov/protein/ETN69481?report=genbank&log$=taxrep&RID=5MZ5BRS0014) | NECAME_15273 | Nam-EPIC-x | **144** | 2 |
| 51 | *Nippostrongylus brasiliensis* | 97 | [A0A0N4XVM2 (NIPBR)](https://www.uniprot.org/uniprot/A0A0N4XVM2) | [VDL70449](https://www.ncbi.nlm.nih.gov/protein/VDL70449?report=genbank&log$=taxrep&RID=6Z06XPZJ016) | NBR_LOCUS6860 | Nbr-EPIC-y | 522 | 6 |
| 52 | *Oesophagostomum dentatum* | 98 | [A0A0B1T3H3 (OESDE)](https://www.uniprot.org/uniprot/A0A0B1T3H3) | [KHJ90661](https://www.ncbi.nlm.nih.gov/protein/KHJ90661?report=genbank&log$=taxrep&RID=5MZ5BRS0014) | OESDEN_09494 | Nbr-EPIC-y | 232 | 2 |
| 53 | *Pristionchus pacificus* | 99 | [H3DXI4 (PRIPA)](https://www.uniprot.org/uniprot/H3DXI4) | [PDM72425](https://www.ncbi.nlm.nih.gov/protein/PDM72425?report=genbank&log$=taxrep&RID=6Z06XPZJ016) | PRIPAC_38859 | Ppa-EPIC-x | 1134 | 13 |
|  |  | 100 | [A0A2A6C2N0 (PRIPA)](https://www.uniprot.org/uniprot/A0A2A6C2N0) | n/a | WBGene00091683 | Ppa-EPIC-z | 396 | 6 |
| 54 | *Teladorsagia circumcincta* | 101 | [A0A2G9UVC1 (TELCI)](https://www.uniprot.org/uniprot/A0A2G9UVC1) | [PIO74195](https://www.ncbi.nlm.nih.gov/protein/PIO74195?report=genbank&log$=taxrep&RID=6Z06XPZJ016) | TELCIR_03802 | Tci-EPIC-x | **343** | 6 |
|  |  | 102 | [A0A2G9UVC8 (TELCI)](https://www.uniprot.org/uniprot/A0A2G9UVC8) | [PIO74194](https://www.ncbi.nlm.nih.gov/protein/PIO74194?report=genbank&log$=taxrep&RID=5MZ5BRS0014) | TELCIR_03801 | Tci-EPIC-z | 247 | 4 |

^1^ Blast was carried out with part of the *Asu-epicut1* nucleotide sequence or the Asu-EPIC-1 conserved peptide (SSGYRKKRNAYGDE) as a query

^2^ Protein names are given in abbreviating the species name into three letters, combined with EPIC-x, EPIC-y, EPIC-z to indicate sequence differences. In clade III EPIC-1, EPIC-2, and EPIC-3 are used for distinct epicuticlins of species described in more details in the manuscript text.

^3^ In clade V red numbers indicate sequences with a cysteine residue in the repeats called EPIC-x.
