# Supplementary material for: Identification and characterization of epicuticular proteins of nematodes sharing motifs with cuticular proteins of arthropods
Source: PLoS One. 2022 Oct 27;17(10):e0274751. doi: 10.1371/journal.pone.0274751 (PMC9612446; doi:10.1371/journal.pone.0274751)
Supplement: Attachment — Submitted filename: Rebuttal document 31 8 2022.docx [file pone.0274751.s007.docx]

Response to reviewers 31 8 2022:

| **Academic editor:** | **Author’s comments** |
| --- | --- |
| A rebuttal letter that responds to each point raised by the academic editor and reviewer(s). You should upload this letter as a separate file labeled 'Response to Reviewers'. | Is provided |
| A marked-up copy of your manuscript that highlights changes made to the original version. You should upload this as a separate file labeled 'Revised Manuscript with Track Changes'. | Is provided |
| An unmarked version of your revised paper without tracked changes. You should upload this as a separate file labeled 'Manuscript'. | Is provided |
| **Journal requirements:** |  |
| Please review your reference list to ensure that it is complete and correct. If you have cited papers that have been retracted, please include the rationale for doing so in the manuscript text or remove these references and replace them with relevant current references. Any changes to the reference list should be mentioned in the rebuttal letter that accompanies your revised manuscript. If you need to cite a retracted article, indicate the article’s retracted status in the References list and also include a citation and full reference for the retraction notice. | Has been verified. As far as we know, there are no retracted papers. No changes were made to the reference list |
| Reviewer #1: The authors have adequately addressed my previous comments. However, their discussion of two epicuticlins in C. elegans does not seem to match what is currently annotated in WormBase. There are three C. elegans epicuticlins annotated with different nomenclature than that used by these authors: EPIC-1, EPIC-2, and EPIC-3. The annotations appear to be relatively recent (2022), so likely this is a new development since the authors began their study. I recommend updating the references to C. elegans epicuticlin genes and adopting the curated nomenclature in WormBase to minimize confusion. | To comply with the Wormbase nomenclature, recommended by Prof T. Schedl, whom we contacted, we decided to change our names from “epicut” to “epic” for all epicuticlin genes described in the manuscript, including the supporting information. An explanatory phrase was added in the M&M section.  The recent annotation of the *Cel* *epic-1-3* genes was cited in the discussion (lines 522-524). Nevertheless, we did not adopt the numbering 1 – 3, since there is no scientific evidence given for the numbering. It seems to be random. We used x, y, and z instead for different epicuticlins of most species to avoid confusion. Asu-EPIC-1 to 3 have unique protein properties, which allow the numbering.  *Asu-epic-1* and *2* are, for example, very different from *Cel-epic-1* and *2* and cannot be related directly. NCBI Blast with *Asu-epic-1* as a query is not able to detect *Cel-epic-1.* |
